# Training Primary Care Physicians in Dermoscopy for Skin Cancer Detection: a Scoping Review

**DOI:** 10.1007/s13187-019-01647-7

**Published:** 2019-12-02

**Authors:** Jonathan A. Fee, Finbar P. McGrady, Cliff Rosendahl, Nigel D. Hart

**Affiliations:** 1 Centre for Medical Education, School of Medicine, Dentistry and Biomedical Sciences, Queen’s University Belfast, Whitla Medical Building, 97 Lisburn Road,, Belfast, BT9 7BL Northern Ireland; 2 School of Clinical Medicine, The University of Queensland, Brisbane, Australia; 3 School of Medicine, Tehran University of Medical Sciences, Tehran, Iran

**Keywords:** Dermoscopy, Primary Health Care, General Practice, Melanoma, Skin Cancer, Continuing Medical Education

## Abstract

In many countries, patients with concerning skin lesions will first consult a primary care physician (PCP). Dermoscopy has an evidence base supporting its use in primary care for skin cancer detection, but need for training has been cited as a key barrier to its use. How PCPs train to use dermoscopy is unclear. A scoping literature review was carried out to examine what is known from the published literature about PCP training in dermoscopy. The methodological steps taken in this review followed those described by Arksey and O’Malley, as revised by Levac *et al*. Four electronic databases were searched for evidence published up to June 2018. Sixteen articles were identified for analysis, all published since 2000. Ten training programs were identified all of which addressed dermoscopy of pigmented skin lesions, among other topics. Ten articles reported on a range of outcomes measured after training and showed generally positive results in terms of improved diagnostic performance, although no meta-analysis was conducted. However, it was unclear whether trained PCPs continued to use dermoscopy after training. Observational questionnaire data revealed that many PCPs use dermoscopy in practice without any formal training. The literature generally supports the use of dermoscopy by PCPs, but it is unclear whether current training leads to long-term change in PCPs’ use of dermoscopy in clinical practice. Understanding this problem, as well as exploring PCPs’ training needs, is essential to develop training programs that will facilitate the uptake and use of dermoscopy in primary care.

**Electronic supplementary material:**

The online version of this article (10.1007/s13187-019-01647-7) contains supplementary material, which is available to authorized users.

## Introduction

Patients in many countries with new or changing skin lesions will first consult a primary care physician (PCP), commonly called a family physician or general practitioner. Skin disease makes up a significant proportion of PCP workload; for example, it is estimated that up to 20% of PCP consultations in the UK relate to the skin [1] and referrals of suspected skin cancers from PCPs to specialist services have risen dramatically in recent years [2]. Dermoscopy has been shown to be an effective tool for the detection of melanoma and the triage of other pigmented skin lesions in primary care [3–5]. However, these improvements in diagnosis are only achieved after training [6], and lack of training has been cited by PCPs in observational studies as a key barrier to the use of dermoscopy [7, 8]. Given the potential of dermoscopy to improve skin cancer detection in primary care, it is crucial to understand how to train PCPs in dermoscopy, and to highlight where the evidence base is currently insufficient, in order to direct future research.

Scoping literature reviews have become an increasingly common approach used to summarize and report the existing evidence in published literature [9]. Scoping reviews are similar to systematic reviews in that they use rigorous and explicit methods that should allow reviews to be replicated. However, they differ from systematic reviews in that they aim to map the main concepts, sources and types of evidence that exist in an area of research, rather than synthesizing the best available evidence to answer a specific question [10]. A scoping review presents, in narrative, tabular or diagrammatic form, an account of results from studies with a wide range of study designs. However, it does not formally appraise the quality of the primary studies. It is therefore a useful methodology where the aim is to understand and summarize the extent of research in a given area where the body of evidence is heterogeneous in nature [9, 10]. Given the relatively unexplored area of dermoscopy training for PCPs, a scoping review was undertaken with the aim of examining current published evidence and to identify where evidence may be currently insufficient.

## Methods

### Research Team

The research team consisted of a general practice specialty trainee (JAF), and three PCPs (FPM, CR and NDH) involved in clinical practice, teaching and medical education research, all of whom have previous experience in scoping literature reviews. JAF, FPM and NDH contributed to the conception of the scoping review. JAF screened articles, and JAF and NDH reviewed full texts for study selection. All the authors contributed to the collation and reporting of the results.

### Research Ethics

Ethical approval was not required for this work, as a secondary analysis of published literature within the public domain.

### Methodological Framework

Methodological frameworks for conducting scoping reviews have been published in the literature. Arksey and O’Malley developed a framework which was subsequently refined by Levac *et al*. [10, 11]. This was the framework followed in this review, as outlined in the following steps.

### Step 1: Identifying the Research Question

Training is recognised as a significant barrier to dermoscopy use in primary care, and the aim of this review was to broadly investigate dermoscopy training for PCPs. For this reason, an open and inclusive question was formed: *What can be known from the literature about how PCPs train in dermoscopy?*

### Step 2: Identifying Relevant Studies

Initial informal literature searches were carried out to identify the various terms used in the literature for dermoscopy, PCPs and training. Previous work carried out by the research team had helped to refine some of the search terms. The expertise of a medical librarian was sought to ensure that there was adequate coverage of relevant databases for formal literature searches.

Formal literature searches were undertaken between June and July 2018. Four electronic databases were searched: Embase, MEDLINE, Scopus and Web of Science. Search terms were altered very slightly between databases to allow for differences in database subject headings (see Supplementary Table 1 for Embase search strategy). Relevant articles from previous work conducted by the research team were also screened.

### Step 3: Study Selection

Citations identified in database searches had their abstracts screened by JAF. Where this was insufficient to make a decision about selection, the whole article was read, but if there was uncertainty, the article was referred for full-text assessment for eligibility. As is standard in scoping review methods, articles available only in the form of conference abstracts were excluded at this stage. Articles written in languages other than English were also excluded.

At this stage two reviewers, JAF and NDH met to discuss the articles. Both reviewers read the full-text articles and considered them for inclusion according to pre-determined inclusion and exclusion criteria (Table 1). Any discrepancies in opinion between the reviewers were resolved by discussion and agreement reached.

**Table 1:** Inclusion and exclusion criteria for article selection

Inclusion criteria: • Studies examining some aspect of dermoscopy training aimed at PCPs. • Participants, if applicable, were mainly PCPs or specialty trainees (or in countries where the term primary care is not in common use, physicians working in a generalist community setting to whom patients self-refer). Exclusion criteria: • General reviews of dermoscopy not focused on primary care. • Commentaries, editorials or letters discussing other articles. • PCPs working in a specialist or secondary care setting. • PCPs participating in screening programs. • Teledermoscopy studies in which dermatologists interpret the images. • Dermoscopy interpreted by artificial intelligence.	

Irrespective of whether articles were included in the final review analysis, the reference lists of all articles reaching this stage were searched, and additional new citations screened by JAF. Any additional articles that passed the screening stage also had their reference lists searched in an iterative process, until no further new citations were generated that reached the full-text assessment stage.

### Step 4: Charting the Data

JAF created a data extraction spreadsheet using Microsoft® Excel (Microsoft, Redmond, USA) and populated it with details of the included papers. Extracted data included authors, year of publication, origins of the research, study design, content and mode of delivery of training interventions, outcome measures and key findings relating to the review question. The corresponding author of one article was contacted to clarify some of their findings [12].

### Step 5: Collating, Summarizing and Reporting the Results

Guidance published by members of the Joanna Briggs Institute and Joanna Briggs Collaborating Centres was used in reporting the results [13]. This included the classification of results under main conceptual categories such as ‘delivery format’ for dermoscopy training programs and ‘previous dermoscopy training’. It also included use of a flowchart to present the literature search and study selection process, and Preferred Reporting Items for Systematic Reviews and Meta-Analyses (PRISMA) guidance was adopted for this purpose [14].

## Results

**Fig. 1:**
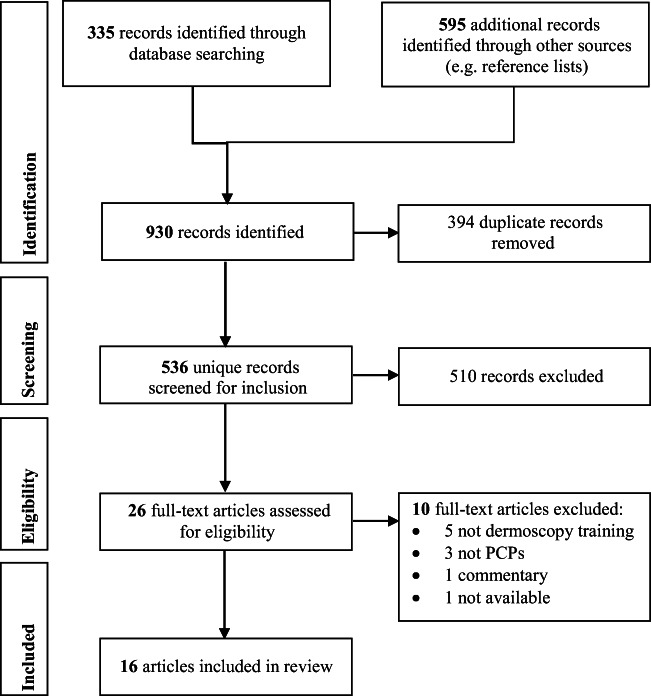
PRISMA flowchart of study selection process [13]

As shown in Fig. 1, 335 citations were identified from database searches, which were carried out sequentially. The fourth database (Embase) produced only two new citations after the exclusion of conference abstracts, neither of which passed the screening stage, and database searches were deemed to be sufficient. Reference lists identified a large number of additional records; however, very few were relevant to the scoping review, and only three articles identified in this way were included in the review analysis.

### Study Design

Sixteen articles were included in the review analysis, comprising a range of study designs. Three systematic reviews were identified, none of which conducted meta-analysis due to study heterogeneity [3, 15, 16]. Ten articles involved interventional studies, five of which reported on four randomised controlled trials (RCTs) [4–6, 12, 17]; the other five on uncontrolled studies of various designs [18–22]. PCPs were the sole participants reported on in seven of the papers; a small number of physician assistants or doctors in other medical specialties participated in the studies reported in three articles, but these papers were included in the review as PCPs formed the majority of participants [12, 17, 22]. One observational questionnaire study was identified [7], and one of the uncontrolled interventional studies also included an observational, questionnaire component [18]. The final two articles were narrative pieces [23, 24].

### Origins of Research

Of the articles selected for analysis, six (38%) originated from Australia; four (25%) from the USA; two each from Italy and the Netherlands and one each from Canada, France and Spain. One study was carried out in two centres in different countries, so was counted towards each country’s total [4]. The first article included in the review was published in 2000 [6], and research in the area has been published steadily since 2005.

### Dermoscopy Training Programs

Eleven articles reported on ten dermoscopy training programs for PCPs. Ten articles reported on interventions that included participants and reported outcomes following the interventions. The 11th article discussed the development of a skin cancer training program for PCPs [23] but did not involve participants. The training programs varied widely in terms of content, delivery format and outcome measures. These were categorized, with categories adapted from those presented by Goulart *et al.* [16] in their systematic review. Definitions of the categories are presented in Supplementary Table 2.

#### Curriculum content

Eight curriculum elements were identified among the training programs, and their inclusion in the different programs is shown in Table 2. All training programs included instruction on skin lesion diagnosis using dermoscopy, and 60% included at least one dermatoscopic algorithm. Seventy per cent also included training on the clinical diagnosis of skin lesions without dermoscopy. All programs trained participants on the differentiation of benign and potentially malignant pigmented skin lesions, but only three included non-pigmented skin lesions. Two studies included training on other diagnostic tools, such as dermatoscopic photography [5] and sequential digital dermoscopy imaging [19]. All programs addressed at least three of the elements, and the largest number of elements addressed in any one training program was seven, as detailed in Table 2.

**Table 2:** Characteristics of dermoscopy training programs for PCPs

Article	Training curriculum								Delivery format				Outcome measures			
(listed by first author and year of publication and ordered by year of publication starting with the most recent)	Epidemiology	Pigmented lesions	Non-pigmented lesions	Clinical diagnosis	Dermatoscopic diagnosis	Dermatoscopic algorithm	Management	Other diagnostic tools	Live	Literature	E-learning	Self-assessment	Diagnostic performance	Knowledge/skill	Confidence/attitude	System outcomes
Robinson, 2018 [17]^c^		X		X	X	X	X				X	X	X^d^	X^d^		
Robinson , 2018 [12]^c^		X		X	X	X	X				X	X	X^a^		X^a^	
Secker, 2017 [18]		X		X	X		X		X	X	X			X^d^		
Koelink, 2014 [5]	X	X	X	X	X	X		X	X				X			X^b^
Shaikh, 2012 [23]	X	X	X	X	X	X					X	X	Not applicable			
Grimaldi, 2009 [20]		X		X	X				X		X	X	X^d^			
Menzies, 2009 [19]		X			X			X	X	X	X		X^d^		X^d^	
Youl, 2007 [21]	X	X		X	X		X			X			X^d^			
Argenziano, 2006 [4]		X	X	X	X	X			X				X^d^			
Dolianitis, 2005 [22]		X			X	X				X	X	X		X^d^		
Westerhoff, 2000 [6]		X			X	X			X	X				X^d^		

^a^No statistical test reported

^b^No statistical test reported, but reported almost 100% chance of cost-effectiveness with €1000 investment

^c^Two articles reported on the same training program

^d^Statistically significant improvement reported in at least one measure in outcome category

#### Delivery Format

Training was delivered using at least one of four formats. Live training and use of e-learning were the commonest formats used, with six programs utilising each approach. Five programs used literature to deliver content, and four used self-assessment. Seven programs used a combination of at least two formats, and three programs employed three formats to deliver training, as shown in Table 2. Duration of training varied from 1 h to 6 months and was not specified in one study [18]. Live training sessions lasted between 2 and 10 h, although two studies specified that only 50–60% of this time was dedicated to dermoscopy training [4, 5]. Longer training periods were seen in self-directed learning programs, although the study with the longest training period of 6 months did not report the length of time that participants actually spent engaging with training materials [21]. In one study, the time taken to train and complete the post-training test were reported as a combined total [22].

#### Training Outcomes

Ten of the 11 articles assessed participants following training, while one article was solely descriptive [23]. The outcome measures were heterogeneous, which precluded meta-analysis. Seven of the ten studies made a measure of diagnostic performance in the clinical setting, four measured knowledge or skills in a classroom setting using photographs, two assessed confidence or attitudes and one measured cost-effectiveness, a system outcome [5]. One study adopted a pass standard for participants, which was set at 85% [17]. Four studies had outcome measures belonging to two different categories. Eight studies reported statistically significant improvements in at least one outcome measure. Of the remaining studies, one reported on cost-effectiveness of dermoscopy in primary care and a cost-effectiveness acceptability curve showed almost 100% chance of cost-effectiveness with €1000 investment [5]; the other did not report any statistical tests [12].

Duration of follow-up when assessing outcomes after training interventions was reported in nine studies and ranged from 2 days to 19 months, median 6 months. Shorter follow-up periods were generally seen in studies in which participants were followed up to a post-intervention test. Studies with longer follow-up periods were generally trials involving data collection from participants’ clinical practice over a period of months.

### Previous Dermoscopy Training

Three studies explored the previous dermoscopy training undertaken by PCPs. Two observational questionnaire studies found that approximately 16% of PCPs reported having had some training in dermoscopy [7, 18]. Another article reported the provision of dermoscopy training for primary care trainees by their training provider in the form of dermatoscopes and reference materials [24].

One study looking at training among French PCPs [7] showed that over half of dermoscopy users had undertaken no formal training, with the commonest type of formal training reported being from books (21%), individual instruction from a dermatologist (13%), attending a course (8%) or online training (5%). Of those in this study undertaking continued training, the commonest forms were attending seminars (30%) and online training (30%), followed by books (13%), and a combination of forms (13%). The total time spent on dermoscopy training by most PCPs was short: less than 1 day for 50% of respondents. Conversely respondents indicated that they felt 7 days was an acceptable length of training to undertake.

## Discussion

Principal Findings

Assessing skin lesions and detecting skin cancer are important roles of PCPs, and training PCPs to use dermoscopy can help them in this task. This scoping review identified 16 articles that have addressed PCP training in dermoscopy. Three articles were systematic reviews, and 11 articles reported on ten training programs for PCPs. Among these were ten interventional studies; however, variability in study designs and outcome measures precluded any meta-analysis, in keeping with previously published reviews [3, 15, 16].

The majority of programs used more than one format to deliver training, but the commonest formats were live delivery and e-learning. E-learning in a self-study format was considered advantageous where participants had to reach a predetermined standard of competency, as the educational time required to reach that standard varied between students [17]. E-learning formats were also considered helpful in overcoming distance barriers between learners and teachers, and in offering a degree of individualisation of learning to participants [23]. However, while the risk of social isolation of learners using e-learning formats has been acknowledged [23], and attending in-person live teaching remains a popular choice for continuing professional development in dermoscopy among PCPs [7], the influence on PCPs of direct contact with dermoscopy experts or other learners in live formats was not explored in the studies identified in this review. The duration of live teaching in training programs was generally short, similar to the short training that PCPs reported undertaking in an observational questionnaire study [7]. However, evidence that many PCPs are using dermoscopy in practice without any formal training raises questions about the accessibility of training, and the competence of these untrained PCPs [7].

All training programs included the assessment of pigmented skin lesions, while a minority addressed non-pigmented skin lesions. This fits with the findings of an observational questionnaire study by Chappuis *et al*. [7] of French PCPs, which reported that dermoscopy was used more for the assessment of pigmented than for non-pigmented skin lesions. However, it raises the concern that current dermoscopy training may not equip PCPs well to identify non-pigmented skin cancers including amelanotic melanomas. The majority of training programs included instruction on the clinical diagnosis of skin lesions, and several included other areas of background information such as risk factors for skin cancer. This highlights that dermoscopy is an assessment tool used as an adjunct rather than an alternative to PCPs’ routine history and examination of skin lesions, and successful use of it will therefore be conditional on PCPs’ proficiency in these other fundamental clinical skills in skin cancer detection and lesion assessment.

Diagnostic performance in the clinical setting was the most commonly reported outcome in trials, and dermoscopy significantly improved performance in the majority. However only one trial addressed a systems outcome [5]. Trials all had relatively short follow-up periods, so it is not possible to determine what effect, if any, dermoscopy training has had on longer-term use of dermoscopy in clinical practice. Of note, one study reported that none of the participants purchased a dermatoscope to continue to use it in clinical practice after the conclusion of the study, despite the improved outcomes that dermoscopy training led to during the trial [12]. It may be that current programs are not meeting the training needs of participants in order to facilitate on-going use of dermoscopy in independent practice, and it is notable that only one study set a pass standard for participants [17]. However, it must also be acknowledged that training is not the only barrier to dermoscopy use, and others such as equipment costs must also be considered and addressed [7, 8].

### Limitations

Scoping reviews are exploratory, and despite using a rigorous and recognised methodology, other papers of relevance may have been overlooked. Furthermore, it is likely that dermoscopy training programs exist in unpublished forms, for example for online or university dermoscopy courses. However, while these could give further insights into dermoscopy training, it was decided not to search for these as part of this scoping review; these courses are open to healthcare workers in many different specialties and roles, so may not have directly addressed the review question with its focus on PCPs.

Given the high proportion of trials with significant results in this review, we must acknowledge the potential for publication bias to influence our findings. By limiting our review to English language articles for practical reasons, published work in other languages may have been overlooked.

The details of training programs were based on information described in published articles, and there may have been elements of programs that were not mentioned by authors and so were not available to the reviewers. Categorising training programs will have obscured some of the nuanced differences between them but is acceptable in a scoping review where the aim is to describe the range and extent of published evidence.

Focusing on PCP training means that other forms of dermoscopy training in other areas of healthcare may have gone unnoticed. Other specialties such as dermatology may have more established or better-tested forms of training. However, unlike dermatologists, for PCPs, skin lesion assessment forms a minority, though significant part, of their clinical work, and dermoscopy use for PCPs will not be as regular as for dermatologists. PCPs often work in more solitary clinical environments, without close proximity to other dermoscopy users. The recognition of these differences, and an acknowledgement that developing and retaining competencies in dermoscopy may require a distinct approach for PCPs, led to a focus on PCP dermoscopy training in this review.

### Research Gaps

This review has highlighted several important gaps in our current knowledge of dermoscopy training. Firstly, longer-term follow-up is needed to determine whether current dermoscopy training programs influence PCPs’ on-going practice. One study noted poor continuing use of dermoscopy among participants [12], and elucidating the factors that contribute to these translational difficulties more fully is essential in supporting use of dermoscopy in primary care.

The lack of long-term follow-up also means that there has been limited study of the impact of PCP dermoscopy training on wider healthcare systems such as dermatology or hospital skin cancer clinics. This is essential to determine whether dermoscopy should remain the preserve of a small group of PCPs with specialist interest in dermatology and skin cancer, or whether it should be expanded to become a standard assessment tool across primary care, similar to a stethoscope or ophthalmoscope.

Only one study set a standard for a minimum acceptable level of proficiency in dermoscopy [17], and the lack of agreed competency standards for dermoscopy use has been acknowledged in countries with significant PCP communities such as the UK [25]. Defining competency standards for dermoscopy could help to guide development of training, allow comparison of training programs and provide PCPs with an objective benchmark against which to assess their progression and further training needs.

No qualitative work has been identified in the published literature. Qualitative research could be useful in exploring translational problems, could be carried out at little cost and could contribute to a better understanding of PCPs’ perceptions of dermoscopy and dermoscopy training that may help to facilitate uptake of dermoscopy.

## Conclusions

This scoping literature review has demonstrated that dermoscopy training for PCPs is currently highly varied, and published trials generally report positive outcomes of training on clinical care in terms of improved diagnosis of skin lesions. However, PCPs who attend short dermoscopy training programs may not continue to use it longer-term in practice, while conversely, some PCPs are using dermoscopy with no formal training. Given the valuable role of dermoscopy in the detection of skin cancers, further work to better define these problems and to seek timely solutions is essential. In particular, qualitative research could help to clarify PCPs’ training needs and to guide training program development to facilitate uptake of dermoscopy in primary care.

## Electronic supplementary material

ESM 1 (DOCX 29 kb)

ESM 2 (DOCX 30 kb)
