# Supplementary material for: Training Primary Care Physicians in Dermoscopy for Skin Cancer Detection: a Scoping Review
Source: J Cancer Educ. 2019 Dec 2;35(4):643–50. doi: 10.1007/s13187-019-01647-7 (PMC7363668; doi:10.1007/s13187-019-01647-7)
Supplement: Supplementary file 1 — (DOCX 29 kb) [file 13187_2019_1647_MOESM1_ESM.docx]

1. Epiluminescence microscopy/
2. Dermoscop*.mp
3. Dermatoscop*.mp
4. “Epiluminescence microscop*”.mp
5. “Surface microscop*”.mp
6. “Incident light microscop*”.mp
7. General practice/
8. “General practice*”.mp
9. General practitioner/
10. “General practitioner*”.mp
11. GP*.mp
12. Family medicine/
13. “Family medic*”.mp
14. “Family practice*”.mp
15. “Family physician*”.mp
16. “Family practitioner*”.mp
17. Primary medical care/
18. “Primary medical care*”.mp
19. Primary health care/
20. “Primary health care*”.mp
21. Primary healthcare*”.mp
22. “Primary care*”.mp
23. “Primary care physician*”.mp
24. Education/
25. Educat*.mp
26. Learning/
27. Learn*.mp
28. Training/
29. Train*.mp
30. Curriculum/
31. Curricul*
32. Teaching/
33. Teach*.mp
34. Competence/
35. Competenc*.mp
36. Skill/
37. Skill*.mp
38. Course*.mp
39. 1 OR 2 OR 3 OR 4 OR 5 OR 6
40. 7 OR 8 OR 9 OR 10 OR 11 OR 12 OR 13 OR 14 OR 15 OR 16 OR 17 OR 18 OR 19 OR 20 OR 21 OR 22 OR 23
41. 24 OR 25 OR 26 OR 27 OR 28 OR 29 OR 30 OR 31 OR 32 OR 33 OR 34 OR 35 OR 36 OR 37 OR 38
42. 39 AND 40 AND 41

**Supplementary Table 1** Embase search terms.
