# Supplementary material for: Training Primary Care Physicians in Dermoscopy for Skin Cancer Detection: a Scoping Review
Source: J Cancer Educ. 2019 Dec 2;35(4):643–50. doi: 10.1007/s13187-019-01647-7 (PMC7363668; doi:10.1007/s13187-019-01647-7)
Supplement: Supplementary file 2 — (DOCX 30 kb) [file 13187_2019_1647_MOESM2_ESM.docx]

**Curriculum**

**Dermatoscopic diagnosis** Principles of skin lesion recognition with dermoscopy.

**Clinical diagnosis** Principles of skin lesion recognition with the naked eye.

**Dermatoscopic algorithm** Use of a step-wise algorithm to aid the dermatoscopic assessment of skin lesions.

**Pigmented skin lesions** Differentiation of benign and potentially malignant pigmented skin lesions.

**Non-pigmented skin lesions** Differentiation of benign and potentially malignant non-pigmented skin lesions.

**Epidemiology** Background information on skin cancer, skin lesion rates, trends in incidence or mortality, risk factors, etc.

**Management** Formulation of a clinical action plan.

**Other diagnostic tools** Training in additional tools to assess skin lesions.

**Delivery format**

**Live** Face-to-face interaction between instructor(s) and participants.

**E-learning** Use of computer software, multimedia or the internet.

**Literature** Written material issued in physical form.

**Self-assessment** Formative practice tests available to participants.

**Outcome measures**

**Diagnostic performance** Objective assessment of diagnostic ability in a clinical setting e.g. sensitivity, excision rates of benign lesions.

**Knowledge/Skill** Objective assessment of knowledge or skills in a non-clinical or classroom setting e.g. dermatoscopic images.

**Confidence/Attitude** Subjective or objective report of confidence in assessment or diagnosis, attitude towards dermoscopy or reported use of dermoscopy e.g. self-ratings.

**System outcomes** Objective assessment of the effects of dermoscopy on patient populations or medical care costs e.g. cost-effectiveness.

**Supplementary Table 2** Definitions of study variables.
